# Impact of elevated serum estradiol levels before progesterone administration on pregnancy outcomes in frozen-thawed embryo transfer for hormone replacement therapy

**DOI:** 10.1186/s12958-024-01260-4

**Published:** 2024-07-30

**Authors:** Jun Shuai, Qiaoli Chen, Siyan Wan, Xingyu Chen, Weiwei Liu, Hong Ye, Guoning Huang

**Affiliations:** 1 Center for Reproductive Medicine, Women and Children’s Hospital of Chongqing Medical University, Chongqing Health Center for Women and Children, No. 64 of Jintang Street, Chongqing, 400013 People’s Republic of China; 2 College of Science, University of Nottingham Ningbo China, Ningbo City, Zhejiang Province People’s Republic of China; 3 Department of Clinical Medicine, Chongqing Medical University, Chongqing, People’s Republic of China

**Keywords:** Hormone replacement therapy, Estradiol, Frozen-thawed embryo transfer, Clinical pregnancy rate, Live birth rate

## Abstract

**Objective:**

The objective of this retrospective cohort study is to investigate the impact of monitoring serum estradiol (E2) levels before progesterone administration within hormone replacement therapy (HRT) on pregnancy outcomes in women undergoing frozen-thawed embryo transfer (FET).

**Methods:**

Analyzed HRT-FET cycles conducted at a reproductive center from 2017 to 2022. Serum E2 levels were measured prior to progesterone administration. Multivariate stratified and logistic regression analyses were performed on 26,194 patients grouped according to terciles of serum E2 levels before progesterone administration.

**Results:**

The clinical pregnancy rate (CPR) and live birth rate (LBR) exhibited a gradual decline with increasing serum E2 levels across the three E2 groups. Even after controlling for potential confounders, including female age, body mass index, infertility diagnosis, cycle category, number of embryos transferred, fertilization method, indication for infertility, and endometrial thickness, both CPR and LBR persistently showed a gradual decrease as serum E2 levels increased within the three E2 groups. The same results were obtained by multivariate logistic regression analysis.

**Conclusions:**

This large retrospective study indicates that elevated serum E2 levels before progesterone administration during HRT-FET cycles are associated with reduced CPR and LBR post-embryo transfer. Therefore, it is advisable to monitor serum E2 levels and adjust treatment strategies accordingly to maximize patient outcomes.

**Supplementary Information:**

The online version contains supplementary material available at 10.1186/s12958-024-01260-4.

## Background

The utilization of frozen-thawed embryo transfer (FET) has witnessed a significant increase, due to the diminished necessity for clinical visits and enhanced scheduling flexibility, hormone replacement therapy (HRT) has found extensive application in FET. Early investigations proposed that employing ultrasound to evaluate endometrial thickness could function as an alternative to invasive methods, such as endometrial biopsy, for gauging endometrial receptivity in the context of FET [1]. Several studies have shown that optimal endometrial thickness is crucial for successful reproductive outcomes in FET cycles. An endometrial thickness of 7–14 mm is generally considered ideal, with thicknesses outside this range being associated with lower pregnancy rates and live birth rates [2–5]. Additionally, reproductive endocrinology is one of the most important regulatory mechanisms in the endometrium, and it is crucial to improve endometrial receptivity by adjusting the application and timing of E2 and progesterone. E2 fosters the proliferation of the endometrium and triggers the expression of progesterone receptors within the endometrial tissue, a prerequisite for the successful implantation of a transferred embryo [6]. A positive correlation exists between serum E2 levels in HRT-FET cycles and the dosage of exogenous E2 medication administered. However, the evaluation of exogenous E2 dosage is influenced by various factors, including the type of drug, administration route, and individual metabolic variations, resulting in significant heterogeneity in the data, posing analytical challenges. Therefore, we opted for serum E2 levels as a research indicator to provide a more precise reflection of the biological utilisation of E2.

Some studies have suggested that supraphysiological levels of serum E2 can disrupt the synchronisation of embryo-endometrial interactions and/or promote pathological endometrial development, thereby impairing optimal endometrial receptivity and embryo implantation [7–9]. However, other research have indicated that elevated supraphysiological concentrations of serum E2 do not impact the physiological levels of progesterone to induce secretory transition [10]. Several investigations have additionally conveyed findings that the serum E2 levels during the mid-cycle phase exhibit no correlation with outcomes in assisted reproductive technology (ART) [11–13]. In fresh embryo transfer cycles following ovarian stimulation, elevated serum E2 levels have been frequently associated with adverse reproductive outcomes, including lower birth weights and preterm births. In contrast, such adverse outcomes are less commonly observed in artificial FET cycles [14, 15]. Elevated E2 levels in fresh cycles are believed to impair endometrial receptivity, potentially due to endometrial overstimulation. This overstimulation can create suboptimal conditions for implantation, leading to poorer pregnancy outcomes. Conversely, in FET cycles, the endometrium is prepared in a more controlled and regulated manner, which may mitigate some of the adverse effects associated with high E2 levels in fresh cycles. However, the need to measure serum E2 levels during artificial cycling remains controversial. Its effect on the final assisted reproductive outcome also remains a subject of debate. Based on the above findings, we aimed to further explore this contentious issue by analyzing a large-scale, single-centre, 6-year retrospective cohort comprising HRT-FET cycles that were uniformly prepared.

## Methods

### Study design and population

In a single-center retrospective cohort study, we collected data from the Centre for Reproductive Medicine’s database for all HRT-FET procedures conducted between January 2017 and December 2022. Throughout this period, embryo cryopreservation exclusively utilized vitrification, and serum E2 levels were assessed using a single hormone assay. Patients were followed up for at least 1 year. Patients with uterine malformations (unicornuate uterus, septate uterus, or double uterus), untreated hydrosalpinx, unaddressed endometrial lesions (endometritis, endometrial polyps, or intrauterine adhesions), those undergoing pre-implantation genetic testing cycles for chromosomal abnormalities, those treated with gonadotropin-releasing hormone agonists, those with embryos derived from in vitro maturation, those who underwent oocyte-donation cycles, and patients who canceled the embryo transfer on the planned transfer day were excluded from the study. Finally, 26,194 patients were included in the study. Patients were classified into three groups based on tertiles of serum E2 level before progesterone administration: group 1 (≤ p33.3, E2 ranging from 10.00 to 118.00 pg/mL, *n* = 8784), group 2 (p33.4–p66.6, E2 ranging from 118.44 to 231.00 pg/mL, *n* = 8662), and group 3 (≥ p66.7, E2 ranging from 232.00 to 3325.00 pg/mL, *n* = 8748). The retrospective study received approval from the Institutional Review Board and Ethics Committee, with waived informed consent due to its retrospective design.

### Artificial FET protocol

All patients underwent HRT for endometrial preparation. Hormonal therapy was initiated on the second or third day of the menstrual cycle after confirming baseline serum hormone levels (E2 < 50 pg/mL, and progesterone < 1 ng/mL). Patients received oral administration of 2–5 mg of E2 (Progynova, Bayer-Schering Pharma AG, Berlin, Germany) twice daily. Transvaginal ultrasound measured endometrial thickness and shape on the seventh day of administration, and E2 dosage adjustments were made based on endometrial thickness, following the physician’s preference and experience. After 12–14 days of treatment, the endometrium underwent re-evaluation using transvaginal ultrasonography, accompanied by measurements of serum E2 and progesterone levels. In instances where endometrial thickness was either < 7 mm or > 15 mm, patients opted for embryo transfer after being informed of associated risks. When serum progesterone levels were < 1 ng/mL, vaginal micronized progesterone (Utrogestan, 200 mg, three times daily; Besins Healthcare, UK) was administered on the second day after completing E2 and progesterone measurements to induce the luteal phase. Embryo transfer for cleavage-stage embryos occurred on the fourth day of progesterone administration, and blastocyst transfer was conducted on the sixth day (the first day of progesterone administration was recorded as D1, cleavage-stage embryo transfer as D4, and blastocyst transfer as D6). An additional daily oral dose of 20 mg dydrogesterone (Duphaston, Abbott, Netherlands) was administered on the day of embryo transfer. Luteal phase support continued until the Human chorionic gonadotropin pregnancy test. If the test result was positive, support extended until the 12th week of pregnancy.

### Definition of clinical outcomes

The primary outcome measures were the clinical pregnancy rate (CPR) and live birth rate (LBR), defined according to the criteria established by the American Society for Reproductive Medicine in 2017 [16]. Clinical pregnancy was ascertained by the presence of one or more gestational sacs detected through ultrasonography. Live birth was defined as the delivery of at least one viable baby after 22 weeks of gestation. The calculation for CPR (%) involved dividing the total number of clinical pregnancy cycles by the number of embryo transfer cycles and multiplying by 100. Similarly, LBR (%) was determined by dividing the total number of live birth cycles by the number of embryo transfer cycles and multiplying by 100.

### Statistical analysis

Participants were initially assigned to distinct groups according to their baseline parameters, and the chi-square test was employed to compare both CPR and LBR. Subsequently, a multivariate logistic regression analysis was conducted to assess the correlation between CPR and LBR. All statistical analyses were carried out utilizing IBM SPSS Statistics 21 (IBM Corp., USA), with all P values being two-sided, and statistical significance set at *P* < 0.05.

## Results

### Characteristics of the patients

The analysis incorporated 26,194 cycles of FET-ART, exhibiting a CPR of 54.4% (14,300/26,194) and a LBR of 44.1% (11,571/26,194). Table 1 summarizes the baseline clinical characteristics and cycle parameters of the participants. Stratifying the cycles into three groups based on tertiles of serum E2 levels revealed a progressive increase in female age, the proportion of patients with secondary infertility, the proportion of cleavage-stage embryo transfers, serum E2 levels both before estrogen administration and before progesterone administration, and the proportion of previous ET or FET cycles with failure. Conversely, body mass index (BMI), the proportion of patients with primary infertility, the proportion of blastocyst transfers, and the proportion of first IVF-FET cycles exhibited a decreasing trend (all *P* < 0.001). In addition, significant differences were observed in the indications for infertility [tubal, endometriosis (EMT), polycystic ovary syndrome (PCOS), and male factor], the number of embryos transferred, and endometrial thickness among the three groups. Significant statistical differences were observed in both CPR and LBR among the three groups (*p* < 0.000 for both), with CPR and LBR decreasing gradually with increasing serum E2 levels (refer to Tables 2 and 3). In addition, Supplemental Table 1 presents detailed descriptive statistics of serum E2 levels before progesterone administration in the three groups, including the mean, standard deviation, range, median, and interquartile range.

**Table 1:** Patients’ baseline characteristics

	Group 1	Group 2	Group 3	*p*
Cycles (*n*)	8784	8662	8748	-
Serum E2 levels before estrogen administration (pg/ml)	31.51 ± 14.72	33.84 ± 15.38	34.00 ± 15.15	0.000**ab
Serum E2 level before progesterone administration (pg/ml)	84.98 ± 23.00	159.74 ± 29.64	794.49 ± 311.53	0.000**abc
Female age (years)	31.81 ± 4.72	32.92 ± 5.12	33.32 ± 5.32	0.000**abc
BMI	22.22 ± 2.95	21.94 ± 2.88	21.90 ± 2.84	0.000**ab
Infertility diagnosis	-	-	-	0.000**
Primary infertility	50.4% (4408/8749)	42.7% (3677/8614)	33.3% (2889/8684)	-
Secondary infertility	49.6% (4341/8749)	57.3% (4937/8614)	66.7% (5795/8684)	-
Cycle category	-	-	-	0.000**
First IVF-FET cycle	44.6% (3921/8784)	40.2% (3482/8662)	33.9% (2964/8748)	
Previous ET or FET cycles with failure	55.4% (4863/8784)	59.8% (5180/8662)	66.1% (5784/8748)	
Stage of embryo transfer	-	-	-	0.000**
Cleavage-stage embryo transfer	75.1% (6598/8784)	77.0% (6669/8662)	78.8% (6894/8748)	-
Blastocyst transfer	24.9% (2186/8784)	23.0% (1993/8662)	21.2% (1854/8748)	-
Number of embryos transferred	1.89 ± 0.35	1.87 ± 0.38	1.87 ± 0.40	0.012*ab
Insemination method-fresh cycle	-	-	-	0.327
IVF	78.8% (5869/7450)	79.0% (5623/7120)	79.7% (5593/7014)	-
ICSI	21.2% (1581/7450)	21.0% (1497/7120)	20.3% (1421/7014)	-
Indication for infertility	-	-	-	-
Tubal	67.0% (5884/8784)	64.1% (5556/8662)	65.4% (5725/8748)	0.000**
EMT	15.7% (1376/8784)	14.6% (1266/8662)	13.0% (1133/8748)	0.000**
PCOS	7.8% (685/8784)	10.8% (936/8662)	13.3% (1166/8748)	0.000**
Male factor	8.2% (720/8784)	7.0% (609/8662)	4.9% (428/8748)	0.000**
Multiple factors	16.7% (1465/8784)	17.1% (1481/8662)	17.4% (1523/8748)	0.434
Endometrial thickness (mm)	8.32 ± 1.21	8.50 ± 1.2	7.79 ± 1.35	0.000**abc

*E2* estradiol, *BMI* body mass index, *IVF* In vitro fertilization, *FET* frozen-thawed embryo transfer, *ET* embryo transfer, *ICSI* Intracytoplasmic sperm injection, *EMT* endometriosis, *PCOS* polycystic ovary syndrome. Different superscript letters (a, b, c) denote significant differences in the pairwise comparisons of the three E2 groups (*P*< 0.05 using Bonferroni correction). 1vs.2 a, 1vs.3 b, 2vs.3 c

**Table 2:** CPR in HRT-FET cycles based on different parameters

	Clinical pregnancy rate						*p*
	Group 1 (8,784)	*p*	Group 2 (8,662)	*p*	Group 3 (8,748)	*p*	
All	59.7% (5242/8784)		56.2% (4869/8662)		47.9% (4189/8748)	0.000**	0.000**
Women Age (years)		0.000**		0.000**		0.000**	
< 35	64.9% (4287/6609)		63.9% (3710/5808)		56.3% (3131/5559)		0.000**
35–39	52.1% (796/1528)		51.5% (921/1790)		42.0% (809/1928)		0.000**
≥ 40	24.6% (159/647)		22.4% (238/1064)		19.7% (249/1261)		0.044*
BMI (kg/m²)		0.089		0.012*		0.000**	
≤ 18.5	69.8% (421/603)		68.7% (483/703)		62.0% (427/689)		0.004**
18.6–24.9	66.3% (3646/5499)		63.5% (3399/5350)		54.9% (2890/5264)		0.000**
≥ 25	64.7% (860/1329)		62.1% (644/1037)		51.7% (531/1027)		0.000**
Infertility diagnosis		0.000**		0.000**		0.000**	
Primary infertility	63.2% (2785/4408)		62.2% (2288/3677)		53.4% (1542/2889)		0.000**
Secondary infertility	56.1% (2437/4341)		51.6% (2549/4937)		45.0% (2606/5795)		0.000**
Cycle category		0.000**		0.000**		0.000**	
First IVF-FET cycle	63.2% (2479/3921)		60.7% (2114/3482)		52.8% (1565/2964)		0.000**
Previous ET or FET cycles with failure	56.8% (2763/4863)		53.2% (2755/5180)		45.4% (2624/5784)		0.000**
Stage of embryo transfer		0.000**		0.000**		0.000**	
Cleavage-stage embryo transfer	55.1% (3634/6598)		50.8% (3389/6669)		43.1% (2972/6894)		0.000**
Blastocyst transfer	73.6% (1608/2186)		74.3% (1480/1993)		65.6% (1217/1854)		0.000**
Number of embryos transferred		0.000**		0.000**		0.000**	
One embryo	47.3% (518/1095)		45.0% (572/1270)		38.9% (523/1346)		0.000**
Two embryos	61.8% (4697/7599)		58.6% (4244/7238)		50.1% (3616/7220)		0.000**
Three embryos	30.0% (27/90)		34.4% (53/154)		27.5% (50/182)		0.385
Indication for infertility							
Tubal	64.5% (3794/5884)	0.000**	62.1% (3452/5556)	0.000**	53.3% (3053/5725)	0.000**	0.000**
EMT	59.7% (822/1376)	0.959	52.9% (670/1266)	0.011*	42.7% (484/1133)	0.000**	0.000**
PCOS	71.5% (490/685)	0.000**	71.5% (669/936)	0.000**	63.2% (737/1166)	0.000**	0.000**
Male factor	78.2% (563/720)	0.000**	74.1% (451/609)	0.000**	70.3% (301/428)	0.000**	0.010*
Multiple factors	68.1% (998/1465)	0.000**	66.4% (983/1481)	0.000**	58.2% (886/1523)	0.000**	0.000**
Insemination method-fresh cycle	-	0.000**		0.014*		0.012*	
IVF	65.1% (3823/5869)		63.2% (3552/5623)		54.4% (3040/5593)		0.000**
ICSI	70.6% (1116/1581)		66.6% (997/1497)		58.1% (825/1421)		0.000**
Endometrial thickness		0.000**		0.015*		0.000**	
< 7 mm	42.7% (47/110)		46.0% (64/139)		35.1% (446/1271)		0.015*
≥ 7 mm	59.9% (5193/8672)		56.4% (4803/8520)		50.1% (3743/7476)		0.000**

*CPR* clinical pregnancy rate, *HRT-FET* hormone replacement therapy for frozen-thawed embryo transfer, *BMI* body mass index, *IVF* In vitro fertilization, *ET* embryo transfer, *FET* frozen-thawed embryo transfer, *EMT* endometriosis; *PCOS* polycystic ovary syndrome, *ICSI* Intracytoplasmic sperm injection

**Table 3:** LBR in HRT-FET cycles according to different parameters

	Live birth rate						*p*
	Group 1 (8,784)	*p*	Group 2 (8,662)	*p*	Group 3 (8,748)	*p*	
All	49.4% (4337/8784)		46.0% (3981/8662)		37.2% (3253/8748)		0.000**
Women Age (years)		0.000**		0.000**		0.000**	
< 35	54.9% (3631/6609)		54.2% (3148/5808)		45.4% (2524/5559)		0.000**
35–39	40.7% (622/1528)		39.1% (699/1790)		31.0% (597/1928)		0.000**
≥ 40	13.0% (84/647)		12.6% (134/1064)		10.5% (132/1261)		0.160
BMI (kg/m²)		0.001**		0.000*		0.000**	
≤ 18.5	60.7% (366/603)		60.6% (426/703)		52.0% (358/689)		0.001**
18.6–24.9	57.4% (3156/5499)		54.2% (2899/5350)		44.7% (2354/5264)		0.000**
≥ 25	52.7% (700/1329)		50.9% (528/1037)		41.1% (422/1027)		0.000**
Infertility diagnosis		0.000**		0.000**		0.000**	
Primary infertility	52.8% (2326/4408)		52.4% (1925/3677)		42.7% (1235/2889)		0.000**
Secondary infertility	45.9% (1992/4341)		41.1% (2031/4937)		34.3% (1988/5795)		0.000**
Cycle category		0.000**		0.000**		0.000**	
First IVF-FET cycle	58.1% (2278/3921)		56.0% (1949/3482)		47.1% (1395/2964)		0.000**
Previous ET or FET cycles with failure	42.3% (2059/4863)		39.2% (2032/5180)		32.1% (1858/5784)		0.000**
Stage of embryo transfer		0.000**		0.000**		0.000**	
Cleavage-stage embryo transfer	44.6% (2945/6598)		40.5% (2704/6669)		33.0% (2273/6894)		0.000**
Blastocyst transfer	63.7% (1392/2186)		64.1% (1277/1993)		52.9% (980/1854)		0.000**
Number of embryos transferred		0.000**		0.000**		0.000**	
One embryo	38.9% (426/1095)		36.9% (468/1270)		29.0% (391/1346)		0.000**
Two embryos	51.3% (3898/7599)		48.1% (3480/7238)		39.2% (2830/7220)		0.000**
Three embryos	14.4% (13/90)		21.4% (33/154)		17.6% (32/182)		0.374
Indication for infertility							
Tubal	55.0% (3239/5884)	0.000**	52.5% (2917/5556)	0.000**	43.1% (2468/5725)	0.000**	0.000**
EMT	47.8% (658/1376)	0.209	42.7% (541/1266)	0.013*	32.7% (370/1133)	0.000**	0.001**
PCOS	61.2% (419/685)	0.000**	60.1% (563/936)	0.000**	50.2% (585/1166)	0.000**	0.000**
Male factor	68.8% (495/720)	0.000**	65.8% (401/609)	0.000**	63.3% (271/428)	0.000**	0.158
Multiple factors	58.2% (852/1465)	0.000**	56.2% (833/1481)	0.000**	46.8% (713/1523)	0.000**	0.000**
Insemination method-fresh cycle	-	0.000**		0.001**		0.013*	
IVF	55.5% (3256/5869)		53.4% (3002/5623)		44.2% (2471/5593)		0.000**
ICSI	61.8% (977/1581)		58.1% (870/1497)		47.9% (680/1421)		0.000**
Endometrial thickness		0.006**		0.004**		0.000**	
< 7 mm	36.4% (40/110)		33.8% (47/139)		26.4% (336/1271)		0.021*
≥ 7 mm	49.5% (4296/8672)		46.2% (3933/8520)		39.0% (2917/7476)		0.000**

*LBR* live birth rate, *HRT-FET* hormone replacement therapy for frozen-thawed embryo transfer, *BMI* body mass index, *IVF* In vitro fertilization, *ET* embryo transfer, *FET* frozen-thawed embryo transfer, *EMT* endometriosis, *PCOS* polycystic ovary syndrome, *ICSI* Intracytoplasmic sperm injection

### Comparison of CPR differences among the E2 groups

The CPR in the three E2 groups were 59.7% (5,242/8,784), 56.2% (4,869/8,662), and 47.9% (4,189/8,748) for groups 1, 2, and 3, respectively (Table 2). For the intra-group analysis of the three groups, CPR was statistically different across different age groups of women (< 35, 35–39, or ≥ 40 years), infertility diagnosis (primary or secondary infertility), cycle category (first IVF-FET cycle or previous ET or FET cycles with failure), stage of embryo transfer (cleavage-stage embryo or blastocyst transfer), number of embryos transferred (one, two, or three), indication for infertility (tubal, EMT, PCOS, male factor, or multiple factors), insemination method-fresh cycle [In vitro fertilization(IVF) or Intracytoplasmic sperm injection(ICSI)], and endometrial thickness (< 7 mm or ≥ 7 mm). For groups 2 and 3, CPR varied among patients based on BMI (≤ 18.5, 18.6–24.9 or ≥ 25 kg/m²) and indications for infertility-EMT.

In the inter-group analysis, after adjusting for confounding factors stratified by age, BMI, infertility diagnosis, cycle category, stage of embryo transfer, number of embryos transferred (one or two), indication for infertility, insemination method, fresh cycle, and endometrial thickness, CPR gradually decreased with increasing E2 levels among the three groups. However, no difference was observed only in the subgroup of patients who underwent transfer of three embryos (Table 2).

### Comparison of LBR differences among the E2 groups

The LBR in the three E2 groups were 49.4% (4,337/8,784), 46.0% (3,981/8,662), and 37.2% (3,253/8,748) for groups 1, 2, and 3, respectively (Table 3). For the intra-group analysis of the three groups, LBR was statistically different across different age groups of women, BMI, infertility diagnosis, cycle category, stage of embryo transfer, number of embryos transferred, indication for infertility, insemination method-fresh cycle, and endometrial thickness. For groups 2 and 3, LBR varied among patients based on the indication for infertility-EMT.

In the inter-group analysis, after adjusting for confounding factors stratified by age, BMI, infertility diagnosis, cycle category, stage of embryo transfer, number of embryos transferred (one or two), indication for infertility, insemination method, fresh cycle, and endometrial thickness, LBR gradually decreased with increasing E2 levels among the three groups. However, no difference in women aged ≥ 40 years and in those who underwent transfer of three embryos (Table 3).

### Influencing factors of CPR and LBR during the FET cycle

Univariate logistic analysis (Supplemental Table 2) was used to evaluate the effect of each variable on pregnancy outcomes. Generally, insemination method-fresh cycle-ICSI, blastocyst transfer, increased number of transferred embryos, tubal disorder, PCOS, male factor infertility, and multiple factors infertility were positively correlated with the CPR and LBR. In contrast, elevated E2 levels (E2 group 2 and 3), advanced age, BMI, secondary infertility, previous ET or FET cycles with failure, endometrial thickness less than 7 mm, and EMT were negatively correlated with the CPR and LBR.

All CPR- and LBR-related factors were reanalyzed simultaneously using a multivariate logistic regression analysis model with adjusted data (Table 4). Elevated E2 levels (third tertile of E2 level group 3), advanced age, endometrial thickness less than 7 mm, and the indication for infertility-tubal were identified as negative predictors for both CPR and LBR. Conversely, positive predictors for CPR and LBR included insemination method-fresh cycle-ICSI, blastocyst transfer, increased number of transferred embryos, the indication for infertility-PCOS, and male factor infertility. Notably, BMI and previous ET or FET cycles with failure were negative predictors for LBR but not for CPR. Serum E2 levels before estrogen administration, infertility diagnosis (secondary infertility/primary infertility), and indications for infertility—EMT and multiple factors—were not included in the final multivariate logistic regression model due to lack of statistical significance.

**Table 4:** Logistic regression analysis of factors associated with CPR and LBR in HRT-FET cycles

	CPR		LBR	
	Adjusted OR (95% CI)	*P*	Adjusted OR (95% CI)	*p*
E2 Tertile Grouping(2/1)	1.029 (0.958–1.105)	0.431	1.020 (0.953–1.093)	0.565
E2 Tertile Grouping(3/1)	1.323 (1.230–1.423)	0.000**	1.326(1.235–1.424)	0.000**
Serum E2 levels before estrogen administration (pg/ml)	-	-	-	-
Age	1.083 (1.076–1.090)	0.000**	1.078 (1.071–1.085)	0.000**
BMI	-	-	1.020 (1.010–1.030)	0.000**
Infertility diagnosis (Secondary infertility/ Primary infertility)	-	-	-	-
Cycle category (Previous ET or FET cycles with failure /First IVF-FET cycle)	-	-	1.422(1.341–1.508)	0.000**
Insemination method-fresh cycle(ICSI/IVF)	0.904 (0.834–0.979)	0.013*	0.880 (0.815–0.951)	0.001**
Stage of embryo transfer(Blastocyst/Cleavage-stage embryo)	0.512 (0.475–0.551)	0.000**	0.604 (0.563–0.647)	0.000**
Number of embryos transferred	0.617 (0.564–0.674)	0.000**	0.749 (0.685–0.819)	0.000**
Endometrial thickness (< 7 mm/≥7 mm)	1.971 (1.739–2.234)	0.000**	1.841 (1.616–2.096)	0.000**
Indication for infertility- Tubal	1.144 (1.047–1.250)	0.003**	1.100 (1.010–1.198)	0.028*
Indication for infertility- EMT	-	-	-	-
Indication for infertility- PCOS	0.907(0.878–0.937)	0.000**	0.929 (0.901–0.958)	0.000**
Indication for infertility- Male factor	0.924(0.892–0.958)	0.000**	0.924 (0.894–0.955)	0.000**
Indication for infertility- Multiple factors	-	-	-	-

*CPR* clinical pregnancy rate, *LBR* live birth rate, *HRT-FET* hormone replacement therapy for frozen-thawed embryo transfer, *OR* odds ratio, *CI* confidence interval, *E2* estradiol, *BMI* body mass index, *ET* embryo transfer, *FET* frozen-thawed embryo transfer, *IVF* In vitro fertilization, *ICSI* Intracytoplasmic sperm injection, *EMT* endometriosis, *PCOS* polycystic ovary syndrome. “-” indicates that the variable is not included in the model

## Discussion

In this study of 26,194 FET-ART cycles, we observed a CPR of 54.4% and an LBR of 44.1%. The stratification of cycles based on E2 tertiles demonstrated a significant association between elevated serum E2 levels and diminished CPR and LBR. These findings suggest that consideration of E2 levels during FET cycles is essential for optimizing reproductive outcomes.

Prior investigations have suggested that increased serum E2 levels during the ovulation stimulation cycle could adversely impact pregnancy outcomes [17–19]. Fritz’s study revealed a substantial decrease in ongoing pregnancy and LBR within the high E2 level group (692–1713 pg/mL) in contrast to the low E2 level group (135–214 pg/mL) [20]. Animal model studies have shown that elevated doses of exogenous E2 may facilitate the shift of the endometrium from a receptive to a non-receptive state. This process is mediated through the rapid closure of the endometrial implantation window, leading to a diminished pregnancy rate [21]. Patel et al. reported a concentration-dependent effect of E2 on protein expression. Specifically, pro-apoptotic caspases 3, 8, and 9 exhibited an increase in expression levels when E2 concentrations reached ≥ 25 nM. Concurrently, the anti-apoptotic protein B-cell lymphoma 2-alpha demonstrated a decline in expression at E2 concentrations exceeding 10 nM [22]. These findings suggest that elevated E2 levels induce trophoblast cell death or impede proliferation. Our study aligns with these precedents, suggesting that increased serum E2 levels before progesterone conversion during HRT cycles may detrimentally affect endometrial receptivity, leading to decreased CPR and LBR. However, in 2020, a retrospective analysis indicated that pre-progesterone E2 levels had no effect on LBR during HRT-FET-assisted reproduction [23]. It has also been shown that pregnancy outcomes are not correlated with serum E2 levels on the day of progesterone conversion in HRT-FET cycles [12, 13, 24]. We identified variations in the definition of elevated E2 levels during HRT cycles across different studies. Serum oestrogen levels are influenced by factors, such as the type of oestrogen medication, administration route (oral, transdermal, or vaginal), and individual metabolic variations, leading to significant interindividual differences and diverse study outcomes. To address this, our study, which was characterised by a substantial sample size, classified serum E2 levels into tertiles and investigated the correlation between serum E2 level trends and assisted reproduction outcomes. This approach effectively addressed potential biases arising from differences in E2 cut-off values and inter-group sample volumes, thereby enhancing the reliability of our results.

In a retrospective study, it is imperative to acknowledge that variations in demographic characteristics among diverse populations can potentially impact research outcomes. Influencing factors in this study include female age, BMI, infertility diagnosis, cycle category, embryo transfer stage, number of embryos transferred, indications for infertility and endometrial thickness, among others. To mitigate the influence of these confounding variables, we conducted multifactorial stratified and multivariate regression analyses. Detailed intra- and inter-group analyses revealed the impact of various clinical parameters on CPR and LBR within each E2 tertile group, revealing multifaceted associations with demographic and clinical factors. Age, infertility diagnosis, stage of embryo transfer, number of embryos transferred, indication for infertility, and insemination method (fresh cycle and progesterone-induced endometrial thickness) emerged as critical factors influencing CPR and LBR, consistent with findings from previous studies [25–28]. A meta-analysis revealed a significant reduction in the probability of achieving clinical pregnancy when the endometrial thickness was ≤ 7 mm, as opposed to cases with endometrial thickness of > 7 mm (23.3% vs. 48.1%, odds ratio: 0.42) [29]. This finding aligns with the outcomes observed at our centre. Notably, only 5.80% (1520/26,188) of patients with endometrial thickness of less than 7 mm underwent transfer, primarily because most patients chose to cancel the procedure when the endometrial thickness fell below 7 mm. In addition, we observed that overweight women (BMI ≥ 25 kg/m²) had lower CPR and LBR than women with normal weight, and multivariate regression analysis also revealed BMI as a negative factor for LBR, with substantial support from existing research supporting our findings [30–32]. Compared to the first IVF-FET cycle, previous ET or FET cycle failures were negative predictors for LBR but not for CPR. In patients with prior embryo implantation failures, there may be underlying abnormalities in endometrial receptivity [33]. Additionally, complex multifactorial causes could contribute to the reduced LBR. Notably, the association between E2 level, CPR, and LBR persisted even after adjusting for these confounding factors, emphasising the independent role of E2 in predicting FET outcomes. These findings also underscore the need for personalised approaches tailored to patient characteristics.

Notably, an interesting observation was made from the subgroup analysis. In most cases, as the E2 levels increased, both CPR and LBR gradually decreased. However, no notable distinctions were detected in the subgroups that underwent the transfer of three embryos. In the subgroup of older women (≥ 40 years), the differences in LBR among women with different E2 levels were also not statistically significant. Several factors may have contributed to this finding. First, patients undergoing a single cycle with the transfer of three embryos in our centre often exhibited poor embryo quality, history of repeated implantation failures, and advanced maternal age. These factors could have individually or collectively influenced the impact of serum E2 level on reproductive outcomes. Additionally, the negative impact of advanced age, especially in women aged ≥ 40 years, may be more pronounced on live birth rates, rendering the positive impact of low E2 levels less significant. Therefore, it is necessary to expand the sample size for this population in future studies.

In addition, the conclusions were further supported by multivariate logistic regression analysis, which identified elevated E2 levels (second and third tertiles) as independent negative predictors for CPR and LBR compared to low E2 levels (first tertile). Supraphysiological E2 levels could potentially disrupt the normal estrogen-progesterone equilibrium within the endometrium, resulting in failed endometrial compaction [34]. Elevated oestrogen levels may also lead to a decreased sensitivity of the endometrium to progesterone, resulting in progesterone resistance. Based on our research findings and reasonable speculation, we consider this to be a promising field for future exploration.

This study has several strengths. Notably, a substantial sample size of 26,194 FET-HRT cycles bolstered the study’s statistical power and enhanced the reliability and generalisability of the findings. In addition to oestrogen, we performed subgroup analyses encompassing various clinical parameters, conducted intra- and inter-group analyses, and employed multivariate regression analyses to optimise the control of confounding factors. Even after adjusting for multiple confounders, our study consistently demonstrated that elevated E2 levels (second and third tertiles) have a potential negative impact on both CPR and LBR compared to low E2 levels (first tertile), offering pertinent guidance for clinical practitioners.

Despite these strengths, this study has several limitations that must be acknowledged. First, its retrospective design introduces an inherent bias. Second, while our study is based on data from a single center, potential unaccounted confounding factors may still influence the final outcomes. For instance, discrepancies in the duration of oestrogen action and measurement time, as well as the specific time interval between ovarian stimulation and FET cycles, may also have affected the results. Although we regret the absence of these data, we assumed that any potential measurement and duration biases were evenly distributed across all study patients, enabling us to analyse the results. Third, the study’s exclusive focus on patients undergoing HRT–FET cycles necessitates caution when generalising the findings to other populations. The outcomes may not be extrapolated to patients undergoing natural or induced ovulation cycles with FET, or to those who opted for IVF with fresh embryo transfer. Therefore, further investigation of these distinct patient cohorts is required.

## Conclusions

This large retrospective study indicates that elevated serum E2 levels before progesterone administration during HRT-FET cycles are linked to reduced CPR and LBR post-embryo transfer. Therefore, it is advisable to monitor serum E2 levels and consider adjusting treatment strategies based on individual patient’s serum E2 levels to maximize patient outcomes.

### Electronic supplementary material

Below is the link to the electronic supplementary material.

Supplementary Material 1

## Data Availability

No datasets were generated or analysed during the current study.

## Abbreviations

FET: Frozen-thawed embryo transfer
HRT: Hormone replacement therapy
ART: Assisted reproductive technology
BMI: Body mass index
CPR: Clinical pregnancy rate
LBR: Live birth rate
E2: Estradiol
EMT: Endometriosis
PCOS: Polycystic ovary syndrome
IVF: In vitro fertilization
ICSI: Intracytoplasmic sperm injection
