# Supplementary figures and images for: Impact of elevated serum estradiol levels before progesterone administration on pregnancy outcomes in frozen-thawed embryo transfer for hormone replacement therapy
Source: Reprod Biol Endocrinol. 2024 Jul 30;22:88. doi: 10.1186/s12958-024-01260-4 (PMC11290307; doi:10.1186/s12958-024-01260-4)

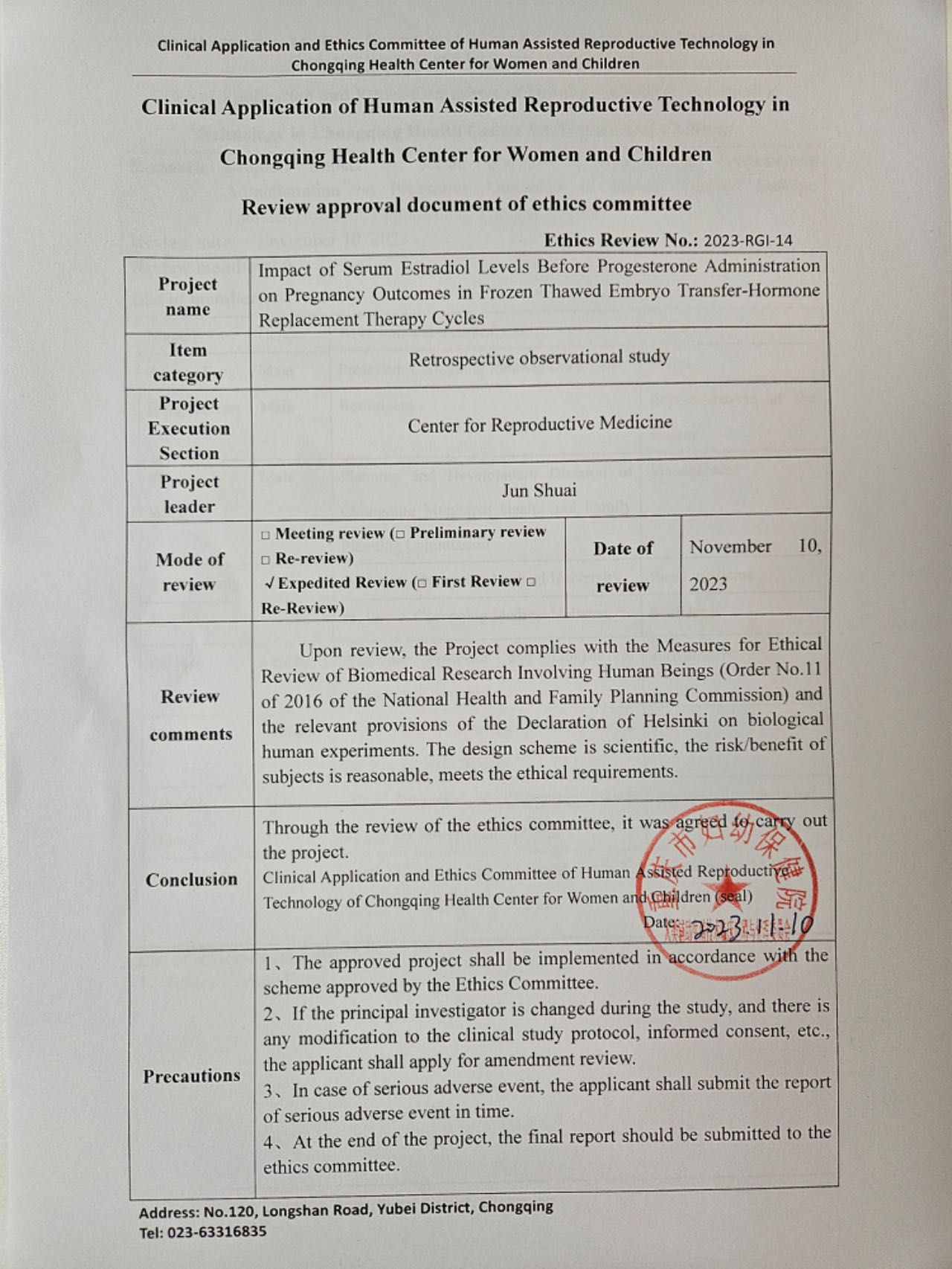

Supplement: Supplementary file 1 — Supplementary Material 1 [file 12958_2024_1260_MOESM1_ESM.jpeg]
